# Comprehensive characterization of neuroblastoma cell line subtypes reveals bilineage potential similar to neural crest stem cells

**DOI:** 10.1186/1471-213X-9-12

**Published:** 2009-02-12

**Authors:** Sandra Acosta, Cinzia Lavarino, Raquel Paris, Idoia Garcia, Carmen de Torres, Eva Rodríguez, Helena Beleta, Jaume Mora

**Affiliations:** 1 Developmental tumor biology laboratory, Hospital Sant Joan de Déu. Passeig Sant Joan de Déu, 2, 08950 Esplugues de Llobregat, Barcelona, Spain

## Abstract

**Background:**

Neuroblastic tumors (NBT) derive from neural crest stem cells (NCSC). Histologically, NBT are composed by neuroblasts and Schwannian cells. In culture, neuroblastic (N-), substrate-adherent (S-) and intermediate phenotype (I-) cell subtypes arise spontaneously.

**Methods:**

Here, neuroblastoma (NB) cell line subtypes were characterized according to embryonic peripheral nervous system development markers (GAP43, Phox2b, Sox10, c-kit, GD2, NF68, vimentin, S100β, calcyclin and ABCG2), morphological features, gene expression and differentiation potential. I-type cells were investigated as a bipotential (neuronal and glial) differentiation stage.

**Results:**

Positive immunostaining of NCSC (GAP43, c-kit, NF68, vimentin and Phox2b) and undifferentiated cell (ABCG2) markers was observed in all NB subtypes. N- and I-type cells displayed cytoplasmic membrane GD2 staining, while nuclear calcyclin was restricted to S-type. N- and I-type cells showed similar phenotype and immunoreactivity pattern. Differential gene expression was associated with each cell subtype. N- and I-type cells displayed similar differentiation capacity towards neuronal and glial lineage fates. S-type cells, upon induction, did not show a neuronal-like phenotype, despite gene expression changes.

**Conclusion:**

Results suggest that N- and I-type NB cell subtypes represent an immature bilineage stage, able to progress towards neuronal and glial fates upon induction of differentiation. S-type cells appear irreversibly committed to a glial lineage fate.

## Background

Neuroblastic tumors (NBT) are the most common extracranial solid tumors in children. NBTs derive from neural crest stem cells (NCSC) blocked at different stages of the sympathetic nervous system development [1,2]. Histologically, NBT are composed of variable proportion of neuroblasts (neuronal lineage) and Schwannian-like cells (glial lineage). Prior studies have reported that both cell types share chromosomal abnormalities, suggesting a common neoplastic precursor cell [3-5] however; discrepant studies failed to detect genetic alterations in the Schwannian components of NBT [6,7].

The differentiation process of NCSC gives rise to peripheral nervous system (PNS), amongst other structures [8,9]. This process can be tracked by analyzing marker proteins sequentially expressed during embryonic cell development [10]. Likewise, the embryonic origin of neuroblastic tumor cells can be established using NCSC protein markers such as Phox2b and c-kit [11,12] while embryonic glial-lineage markers such as SRY box 10 transcription factor (Sox10) and AP2α are expressed in immature glial cells in NBT [2].

NBT cell components may be identified using a variety of protein markers. The glial component may be identified by the expression of the calcium binding family protein S100. Specifically, S100A6 (Calcyclin), has been reported as a neural crest derived glial-cell lineage marker [13], also expressed in the fetal CNS glia [14]. Calcyclin migrates to the nuclear membrane upon an increase of intracellular calcium concentration; its precise function remains unknown [15]. In NBT, calcyclin expression is mostly found in Schwann-like cells [16].

The neuronal lineage compartment of NBT is characterized by the expression of proteins like neurofilament proteins and the membrane ganglioside GD2 [17-19]. GD2 is expressed in fetal brain neuroblasts and neuroectodermal derived tumors. Cell membrane GD2, synthesized by the GD2 synthase, is implicated in cell adhesion [20].

Biedler [21] described three cell subtypes spontaneously arising in NB cell line cultures, the N (neuroblastic), S (substrate adherent) and I (intermediate) subtypes. These three cell line subtypes were isolated and characterized on the basis of the different cell culture behavior and protein expression pattern [21,22]. I-type cell lines, reported to have a bipotential capacity to differentiate towards both neuronal and glial lineage when induced with retinoic acid and bromodeoxyuridine respectively, have recently been proposed as NBT stem cells [23,24]. Retinoic acid is a vitamin A derived molecule, which plays an important role in the early development of the nervous system stimulating neuronal differentiation and inducing the expression of neuronal proteins [25]. Bromodeoxyuridine is a thymidine analog which incorporates into the newly synthesized DNA during the S phase of cell cycle. It has been used as a glial differentiation factor *in vitro *for NB, inducing cell flattening and increased substrate adhesiveness [23,26,27].

In this study, we aimed to characterize each NB cell line subtype according to differential lineage markers from the embryonic development of PNS and test whether the intermediate subtype of NB derived cell lines reproduce a distinct and bipotential (neuronal and glial) stage of differentiation. A continuum process of differentiation between neuronal and glial lineage, which resemble the normal PNS development, was identified.

## Results

### Cell lines characterization

Eleven cell lines were investigated. In addition to morphological and *in vitro *behavior features, cell lines were characterized based on the differential expression of previously described markers [11,23] using immunofluorescent staining (Table 1) and qRT-PCR analysis.

**Table 1:** Immunophenotypical analysis of neuroblastoma cell lines

		**GD2**	**NF68**	**Calcyclin**	**Phox2b**	**c-kit**	**Vimentin**	**GAP43**	**ABCG2**
N-type	LA1-55N	M^++^	C^+^	C	N	C^+^	C^++^	C^+^	C^+^
	LAN-1	M^++^	C^+^	C	N/C^+^	C^+^	C^++^	C^+^	C^+^
	SH-SY5Y	M^++^	C^+^	C‡	N	C^+^	C^++^	C^+^	C^+^
	Be2-M17V	M^++^	C^+^	C‡	N	C^+^	C^++^	C^+^	C^+^

I-type	SK-N-JD	M^++^	C^+^	C	N^+^	C	C^++^	C^+^	C^+^
	SK-N-ER	M^++^	C^+^	C‡	N/C^+^	C^+^	C^++^	C	C^+^
	SK-N-Be(2)C	M^++^	C^+^	C	N^+^	C^+^	C^++^	C^+^	C‡
	SK-N-LP	M^++^	C^+^	C‡	N^+^	C^+^	C^++^	C	C^+^

S-type	SK-N-AS	⫲	C	N^++^	N/C^+^	C^+^	C^++^	C	C^+^
	SH-EP1	⫲	C	N^++^	N/C	C	C^++^	C	C
	LA1-5S	-	C	N^++^	N/C^+^	C	C^++^	C	C

M: membrane; C: cytoplasm; N: nuclei; -: no staining; ‡: some nuclei positive; ⫲: some cell membrane positive; +: moderate staining; ++: intense staining

Distinct morphological features were observed for S- and N-type cell lines, as previously described [21]. S-type cell lines were morphologically characterized by large flat cytoplasms, relaxed chromatin and strong adherence to the flask surface. N- and I-type cell lines showed a very similar phenotype in culture, characterized by a low cytoplasm/nuclei ratio, poor adherence to the flask surface, growth in dense cell aggregates and neurite processes formation. Only one cell line displayed a clear intermediate phenotype, the I-type cell line SK-N-ER, characterized by a larger cytoplasm and greater adherence to the flask surface than the other I-type cell lines, features resembling those of the S-type cell lines.

**Figure 1:**
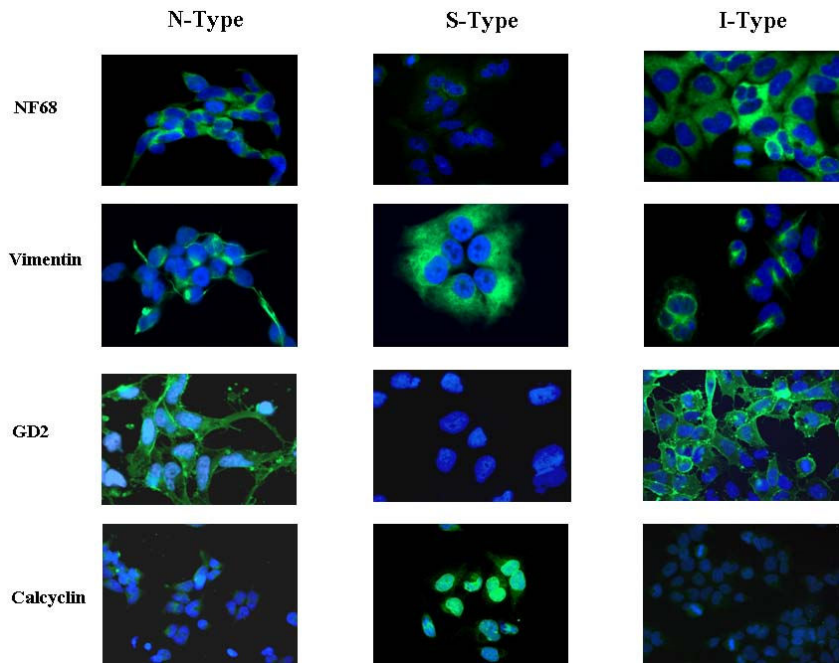
**Neuroblastic and glial immunophenotype in NB cell line subtypes**. Neurofilament NF68 cytoplasm expression intensity was higher in N-, I- than in S-type cell lines. Vimentin is expressed intensely in the cytoplasm of the three cell lines subtypes. GD2 cell membrane and intense nuclear membrane calcyclin staining allows to distinguish between the neuroblastic and the glial phenotype. Blue: DAPI, Green: FITC.

Amongst the NBT cell line subtypes the intermediate neurofilaments and vimentin have been proposed as differentially expressed markers for neuronal and glial phenotypes, respectively [11]. As shown in figure 1, both proteins are expressed in the cytoplasm of the three cell subtypes. The neurofilament NF68 showed a higher cytoplasmic expression in the N- and I cell subtypes (Figure 1). On the other hand, vimentin was detected with similar intensity, in the cytoplasm of all the cell lines analyzed (Figure 1). In our hands, these markers were not helpful to distinguish amongst the cell lines subtypes.

N- and I-type were immunophenotypically distinguishable from S-type cell lines based on the differential staining of GD2 and calcyclin. Similar intense cytoplasm membrane GD2 immunostaining was observed in both N- and I-type cells, while intense nuclear membrane calcyclin was identified exclusively in the S-type. N- and I-type showed a faint diffuse cytoplasm staining for calcyclin (Figure 1). Thus, among the markers tested, we found cytoplasm membrane-GD2 and nuclear-calcyclin immunolabelling as reliable NBT neuroblastic and glial lineage markers, respectively.

Of note, all but one of the S-type cell lines studied, the exception being LA1-5S, turned out not to be pure cultures since few morphologically N-type cells, which were GD2-membrane positive, were identified. S-like cells were also detected in the N-type cell lines Be2-M17V and SH-SY5Y (Table 1).

Overall, the N- and I-type cell lines studied showed a very similar phenotype in culture, with equivalent adherence, cytoplasm/nuclei ratio, and similar neurite process formation. N- and I-type cell lines shared a similar immunoreactivity pattern, namely high membrane GD2 staining, absence of nuclear calcyclin and high neurofilament NF68 (Figure 1).

**Figure 2:**
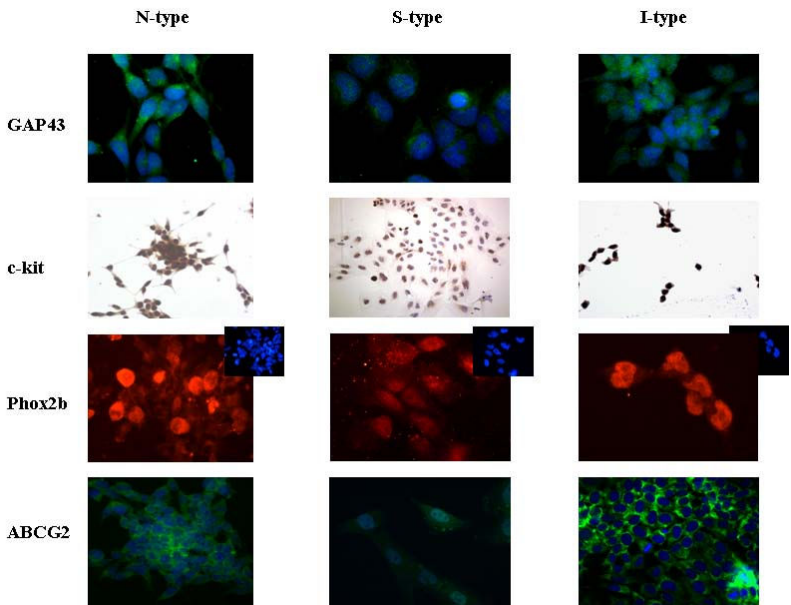
**Neural crest cell markers pattern in NB cell line subtypes**. All cell line subtypes exhibited positive protein expression for GAP43, c-kit, Phox2b and ABCG2 immunostaining. Differences in the intensity and localization of these proteins can be identified in some of the cell lines (see text). For Phox2b, small squares show non-merged nuclear cell images. Blue: DAPI, Green: FITC (ABCG2, GAP43). Red: Cy3 (Phox2b). c-kit (DAB staining).

Protein markers of undifferentiated neural crest derived cells, such as GAP43, c-kit and Phox2b, as well as the undifferentiated cell marker ABCG2, were positive in all the cell line subtypes tested (Table 1). Intense GAP43 immunostaining was detected in the cytoplasm of N-type cell lines (Figure 2) and two of the four I-type cell lines. S-type and the rest of I-type cell lines showed a lower intensity of GAP43 cytoplasmic staining (Table 1). Similar results were observed for c-kit immunostaining (Table 1). On the other hand, nuclear localization of Phox2b positivity was identified in the I-type cell lines, in some cells being very intense. A lower nuclear intensity was identified for the N- and S-type (Figure 2). ABCG2 exhibited a moderate diffuse cytoplasmic immunoreactivity in most of the cell lines analyzed (Figure 2).

**Figure 3:**
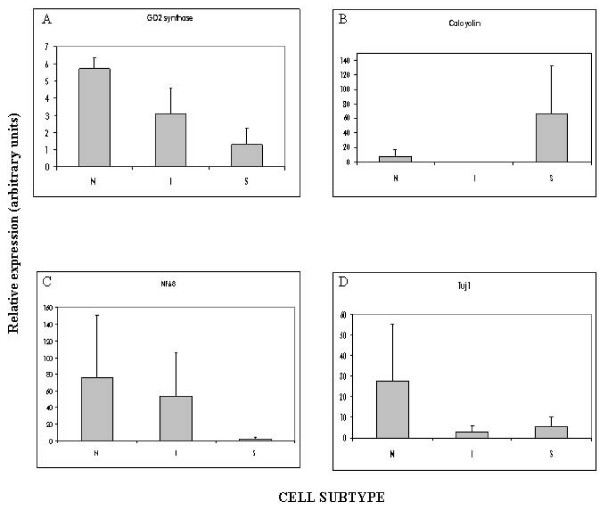
**Gene expression pattern in the NB cell linesubtypes**. Results are displayed as mean expression levels of cell subtypes (N-, I- and S-type), obtained from three independent experiments. Quantification was performed relative to the cell line with the lowest amplification level. Error bars illustrate the variability amongst the cell lines of each subtype. **A**. GD2 synthase mean expression quantified in relation to LA1-5S. **B**. Calcyclin mean expression quantified in relation to LA1-55N. **C**. NF68 mean expression quantified in relation to SK-N-ER. **D**. Tuj1 mean expression quantified in relation to LA1-5S.

To delve further into the differential expression among cell line subtypes, GD2-synthase and calcyclin mRNA expression were analyzed by qRT-PCR. GD2-synthase expression pattern showed differences among the 3 cell subtypes, being the N-type cells those with the highest expression followed by the I-type cells and, at lower level, the S-type cells (Figure 3A). When calcyclin mRNA expression was quantified, different expression for S-type compared to N- and I-type was found (Figure 3B). These results confirm those observed by immunofluorescence.

Additional gene expression analyses were performed in the cell subtypes by qRT-PCR. Neuronal lineage markers, NF68 and Tuj1, showed the highest expression in N-type cells. I-type cells showed higher expression than S-type for the NF68 marker (Figure 3C), and lower for Tuj1 (Figure 3D). Sox10, an immature neural crest marker also expressed in glial cells, did not show detectable expression by qRT-PCR in any of the cell lines analyzed, regardless of the cell type (data not shown).

Altogether, the observed gene expression patterns confirm the inmunofluorescent results underscoring the immature, yet distinct, nature of these NBT cell lines. Nonetheless, I-type cell lines share features closer to the N-type subgroup, namely immature neuronal lineage phenotype, whilst S-type cell lines embody a glial immature stage.

## Differentiation Studies

To investigate the behavior of the different cell subtypes during differentiation induction, LA1-55N (N-type), SK-N-Be2C, SK-N-LP and SK-N-ER (I-type), and LA1-5S (S-type) were chosen to represent each cell subtype.

### I. Neuronal differentiation induced by all-*trans *retinoic acid (ATRA)

#### I.a. Morphological and immunophenotypical changes

**Figure 4:**
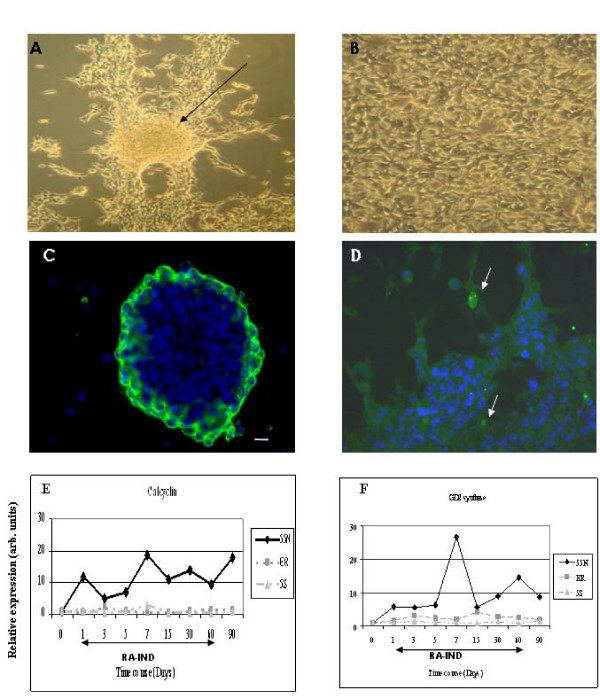
**Morphological, immunophenotypical and gene expression changes induced by ATRA**. Neuronal differentiation in the I-type cell line SK-N-Be2C: **A**. 60 days of ATRA treatment. Arrow: neurosphere in formation. **B**. not-treated negative control culture. **C**. GD2 expression in newly formed floating neurosphere at day 15 of ATRA treatment. **D**. Calcyclin expression at day 60 of ATRA treatment. White arrow: calcyclin positive cell that appear during neuronal differentiation induction treatment. Gene expression changes of calcyclin (**E**) and GD2 synthase (**F**) in LA1-55N, LA1-5S and SK-N-ER cell lines. q-RT-PCR analysis was performed by triplicate of two separate differentiation experiments (60 days induction and 30 days of treatment depletion (90). RA-IND: ATRA induction period.

When differentiation was induced towards neuronal lineage by treatment with ATRA, both N- and I-type cell lines changed their morphology. One week after treatment most cells showed a neuronal-like phenotype, displaying long and growing neurites. These cells proliferated forming large, dense interconnected cell clumps that were eventually released as floating spherical cell aggregates, neurospheres, with capacity to grow in suspension (Figure 4A and 4B). In the N-type, changes appeared early in the first week of treatment and persisted during all the differentiation induction experiment (60 days). All the I-type cells showed morphological changes, the most remarkable being in the SK-N-Be2C cell line. During the first week of treatment neurites grew significantly, and, after two weeks, floating neurospheres appeared. The I-type SK-N-LP cell line did not form neurospheres, but poorly adherent cell clusters. In the I-type SK-N-ER cell line, the morphological changes appeared later and around the 5^th ^week of treatment they formed neurospheres. In all the N- and I-type cell lines, the morphological changes remained evident even when ATRA treatment was removed; however, the capacity to form neurospheres was clearly diminished. Overall, no significant change in the proliferation ratio was detected in the cell lines during treatment, however, an increase in cell mortality was observed after one week of treatment.

Both N- and I-type cell lines showed no variation of the membrane-GD2 immunostaining during ATRA treatment in comparison to non-treated control cell lines. GD2 cell membrane staining was observed in all the newly formed neurospheres (Figure 4C). For S-type cells, no GD2 staining was observed during the entire neuronal induced differentiation experiment. No changes of ABCG2 and NF68 neurofilament immunostaining was detected in the cell lines.

In all the N- and I-type cell lines, some nuclear calcyclin positive cells were detected during ATRA induced neuronal differentiation. S-like cells were initially detected in the periphery of the adhered neuronal clusters (Figure 4D). Throughout the induction of neuronal differentiation, these nuclear-calcyclin positive cells increased in number and acquired S-type morphology, enabling us to isolate them attached to the flask taking advantage of their greater capacity of adherence. However, when isolated from the neuronal cells, these potentially S-type cells stopped growing and died after 2–3 weeks. New aliquots of the cell lines tested were thawed and treated separately to verify whether the presence of nuclear-calcyclin positive cells was not due to cross contamination. Furthermore, non-treated control cell lines were studied in parallel and did not show any of the abovementioned changes observed during the ATRA treatment. Repeated experiments demonstrated that newly induced S-like cells arise in response to ATRA treatment.

The S-type cell line LA1-5S showed no apparent changes when treated with ATRA. It did not show changes in morphology, continued to be adherent to the flask and preserved a flat and large cytoplasm. However, calcyclin nuclear membrane staining decreased during treatment and was re-established upon ATRA removal.

#### I.b. Calcyclin, GD2 synthase and SOX10 gene expression changes

To further investigate changes induced during ATRA differentiation assays, mRNA levels of relevant genes like calcyclin, GD2 synthase and SOX10 were analyzed by qRT-PCR at different time points in the three cell subtypes (LA1-55N, SK-N-Be2C, SK-N-ER and LA1-5S).

ATRA treatment induced an increase of calcyclin transcript levels in the N-type LA1-55N cell line. The I-type cell line, SK-N-ER, displayed a peak of expression (more than 2-fold) at day 3 of treatment (Figure 4E). The S-type cell line, LA1-5S, showed a slow decrease of the calcyclin expression level after 3 days of treatment, with a recover of expression detected at day 7 when ATRA was removed (Figure 4E). In the I-type SK-N-Be2C cell line, calcyclin mRNA expression was undetectable by q-PCR until day 60, when an increase of expression was detected that decreased again to undetectable levels after ATRA depletion (data not shown).

GD2 synthase expression increased during ATRA treatment in N- and I-type cell lines, with a significant increase in the LA1-55N cells. However, LA1-5S showed a minor increase, with a 1.49–0.85 fold variation of expression levels with respect to non-treated controls (Figure 4F).

SOX10 expression was studied in order to clarify the new appearance of S-like cells during neuronal-lineage induced differentiation experiments. SOX10 mRNA was undetectable by q-PCR in all the cell lines tested, both in the treated as well as in the non-treated controls cell lines (data not shown).

Taken together, these results show that I-type and N-type cells display a similar behavior when neuronal differentiation is induced, both showing the capacity to differentiate along the neuronal lineage. On the other hand, for S-type cells, no neuronal-like phenotype was observed, despite the gene expression changes detected. The S-like cells arising during neuronal lineage induced differentiation suggest the bipotential capacity of some of the neuroblastic cells in both N- and I-type cultures.

### II. Bromodeoxyuridine (BrdU) induced glial differentiation

In order to evaluate the capacity of each of the cell lines to differentiate towards glial lineage, BrdU was used as previously described [11,26].

#### II.a. Morphological and immunophenotypical changes

**Figure 5:**
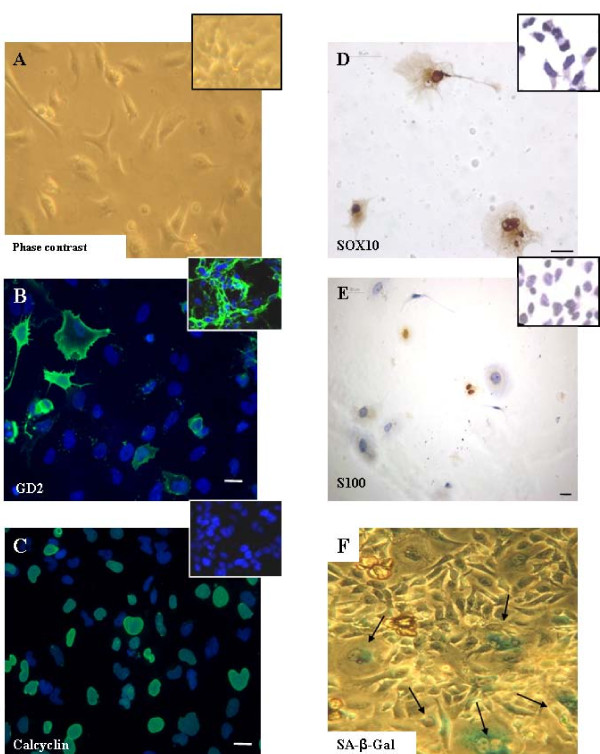
**Morphological and immunophenotypical changes induced by BrdU glial differentiation treatment**. **A**. Morphological changes in SK-N-ER cells (Phase contrast microscopy; 100×). B. GD2 expression levels after 15 days of treatment in I-type cell line SK-N-Be2C. **C **Calcyclin, (**D**) Sox10 and (**E**) S100 expression after 21 days of BrdU treatment in N-type cell line LA1-55N. **F**. SA-β-GAL positive cells (arrow) after 21 days of BrdU treatment in the I-type SK-N-ER (Phase contrast; 100×). Control cell images are reported in the small squares. Scale bar: 50 μm.

After one week of BrdU treatment, morphological changes became apparent. N- and I-type cell lines acquired an S-like morphology, large flat cytoplasm, and augmented adherence to the flask surface (Figure 5A). The S-type cell line did not show any apparent changes. Immunophenotypically, the N- and I-type cell lines showed a decrease of GD2 staining at day 7 of BrdU treatment in comparison to the non-treated control culture (Figure 5B). Calcyclin staining in the nuclear membrane appeared in some cells one week after induction, corresponding with the occurrence of the above mentioned changes (Figure 5C). Likewise, in all the cell lines subtypes, N, S and I, cytoplasmic Sox10 staining increased during treatment and at 3 weeks some nuclear positive cells appeared (Figure 5D). Specifically, S100 staining showed a slight increase during BrdU differentiation, increasing the cytoplasmic staining and showing nuclear staining in some cells after 21 days of treatment (Figure 5E). These changes were not detected in the non treated control cells.

After 3–4 weeks of treatment, all N, I, and S cell lines showed a degenerated morphology, characterized by large cytoplasms, multinucleated cells and a moderate decrease of their growth rate. Narath et al. (2007) [28] have recently described this morphology associated to senescence in neuroblastoma cell lines. In order to test whether S-like induced cells were senescent, SA-β-galactosidase test was performed. A significant increase of senescent cells was observed only at day 21 of BrdU treatment. Specifically, after 15 days of treatment, less than 1% of cells were senescent (SA-β-GAL positive cells) in most of the cell lines studied; the same proportion was observed in non-treated control cells. However, after 21 days of treatment, 50–80% of cells were positive for SA-β-GAL (Figure 5F) in LA1-55N, LA1-5S and SK-N-Be2C and, only 15% of senescent cells were detected in the I-type cell line SK-N-ER.

#### II.b. Calcyclin, GD2 synthase and SOX10 gene expression changes

**Figure 6:**
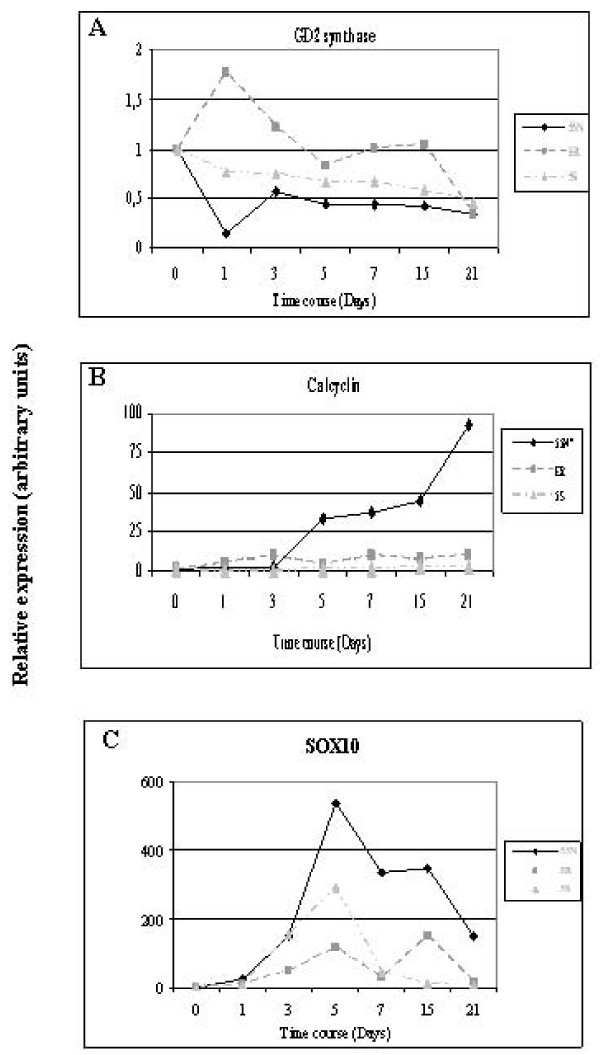
**Quantitative RT-PCR analysis of gene expression changes induced by BrdU treatment in NB cell line subtypes**. Gene expression changes of GD2 synthase (**A**), calcyclin (**B**) and Sox10 (**C**) in LA1-55N, LA1-5S and SK-N-ER cells. q-RT-PCR was performed by triplicate of two separate differentiation experiments (21 days induction).

Gene expression analyses of calcyclin, GD2 synthase and SOX10 (Figure 6) were performed in all 3 cell lines subtypes. A 21-day time course was used for these experiments due to the state of cellular degeneration detected after three weeks of treatment. Days 1, 3, 5, 7, 15 and 21 were analyzed by qRT-PCR.

GD2 synthase expression showed a significant decrease in LA1-55N and LA1-5S, N- and S-type respectively. In, SK-N-ER (I-type), GD2 synthase mRNA level decreased after the 5^th ^day of BrdU treatment, but not as dramatically as for the other cell subtypes (Figure 6A). On the other hand, calcyclin gene expression showed a progressive increase in all the cell lines during treatment. The major increase was observed in the N-type cells. The I-type cell line, SK-N-ER, and the S-type, LA1-5S showed a progressive increase of calcyclin expression (Figure 6B). However, SOX10 showed a great increase of mRNA levels within the first week of treatment, followed by a fast decrease in all the cell lines tested (Figure 6C).

Taken together, BrdU differentiation results suggest the capacity of N- and I-type cell lines to differentiate towards a glial lineage phenotype.

## Discussion

The neural-crest is a vertebrate-specific transient embryonic tissue that gives rise to several cell types [29]. It has been clearly established that early neural-crest cells are multipotent at the cellular level [30], and perhaps in reflection of the innate multipotency, neural-crest derived cells display long lasting plasticity during embryonic development [31]. This plasticity is also reflected in the phenomenon of transdifferentiation, whereby cells change from one unique phenotype into another without going through a developmentally less mature stage [32]. Neural-crest derived tumors like NBT as well as NBT cell lines show many of the cell phenotypes characteristic of the developing neural crest cells: cellular heterogeneity, plasticity and transdifferentiation capacity [24].

Normal embryogenesis of the PNS involves a bilineal stage of cell differentiation whereby early glial and neuronal markers can be found coexpressed [10]. In neuroblastoma, I-type cells have been described to exhibit an intermediate morphological and *in vitro *behavior, sharing characteristics of both N- and S-type cells, and express neuronal as well as glial lineage markers, and to reproduce a distinct and bipotential (neuronal and glial) stage of differentiation [11,24].

Instead of neurofilaments and calcyclin, we found cytoplasm membrane-GD2 and nuclear-calcyclin as reliable NBT neuroblastic and glial lineage markers, respectively. By immunofluorescence, N- and I-type showed similar pattern of expression with high GD2 and undetectable calcyclin. On the other hand, S-type exhibited a high nuclear-membrane calcylin staining and no ganglioside GD2 expression. I-type cells represent thus, a distinct NB cell subpopulation, an intermediate state between the neuronal and glial lineages, however closer to neuroblastic lineage.

In this study, we aimed to characterize each NBT cell line subtype according to differential lineage markers from the embryonic development of PNS. Initially, lineage markers such as vimentin and GAP43 (glial and neuronal, respectively) were studied. In our hands, these proteins did not display differential expression between the different cell subtypes. An undifferentiated state of the cell lines was studied by using neural crest cells markers such as c-kit and Phox2b. These proteins displayed positive immunostaining in all the cell lines subtypes, suggesting a derivative from an immature state, comparable to neural crest cells.

NB cell lines can be induced to differentiate along many neural crest cell lineages like chromaffin cells, Schwann cells, melanocytes and neurons [33,34]. Differentiation inducers towards neuronal lineage like ATRA or glial lineage like BrdU have been thoroughly described in NBT [22,11,35]. We showed that, upon ATRA treatment, both N- and I-type cell lines can be induced to differentiate towards a neuronal-like lineage, while, no neuronal-like phenotype was observed in the S-type cell line, despite the gene expression changes detected.

Furthermore, with BrdU treatment, our results for N- and I-type cell lines showed a similar shift towards a glial-like phenotype (S-type shape, reduced GD2 and increase of glial-lineage markers) for both cell subtypes. Recently, Narath et al. (2007) [28] described cellular senescence in MYCN amplified F-type NBT cells after 6 weeks of hydroxyurea treatment. F-type cells share some morphological features with the S-type cells. In our experiments, the abnormal aspect of BrdU treated cells and proliferation arrest after few weeks of treatment suggested that long exposition to BrdU might induce senescence. Our results, however, demonstrate that BrdU induced *bona fide *glial differentiation for the first two weeks of treatment. After longer exposure (3 weeks) to BrdU, senescence was induced.

In order to further correlate the embryonic development of the PNS and the *in vitro *model, we studied Sox10 induction. Sox10, which is expressed in NCSC and a subset of neural-crest derived lineages, plays a key role in maintaining pluripotency and inhibiting premature differentiation at the stem cell stage. *In vivo*, glial-lineage differentiation is a default program during the NCSC differentiation pathway towards a neuronal fate [10]. Downregulation of SOX10 is a prerequisite for neuronal lineage differentiation and its expression is maintained in the terminally differentiated glial cell [36,10]. Our results show that SOX10 is undetectable in all cell lines and ATRA treatment does not activate SOX10 expression. However, SOX10 expression is induced when glial differentiation is promoted by BrdU treatment with a fast increase of SOX10 expression in the S-type cell line and slower but significant increase in the N- and I-type cell lines.

Our results suggest that N- and I-type NB cells represent an immature bilineage stage, able to progress towards neuronal as well as glial fates upon induction of differentiation.

In addition to neurite elongation, neuron specific protein and gene expression induction, when N- and I-type cell lines were induced to differentiate with ATRA, two phenomena appeared concomitantly: clumps floating spheres similar to the previously described neurospheres [11] and newly formed S-type cells. Neurospheres formation has been previously reported to occur spontaneously in N-type cell lines [11]. The spontaneous or induced formation of neurospheres or big floating cell clusters has been described related to neural stemness [37]. Ongoing experiments in our laboratory are investigating whether the differentiating neurospheres contain multipotent stem-like cells.

Concurrently to neuroblastic cells clustering, S-like cells appeared in the periphery of these cell clusters. Motohashi et al., (2007) [38] have recently reported the presence of glial cells in ATRA treated NCSC cultures. Neuron-glia interactions control several processes in brain development, such as neurogenesis, myelination, synapse formations and neuronal migration, proliferation and differentiation [39,40]. During PNS development neuronal and glial differentiation are interdependent and the fates of these two types of cells are inextricably entangled. Moreover, in neuroblastoma, it has been hypothesized that Schwann cells release factors that could induce neuronal differentiation, providing better prognosis [41]. Our results, first the appearance of S-like cells during induction of differentiation with ATRA in N- and I-type cells, and second the incapacity of these cells to survive devoid of N-/I-type cells, suggest a necessary cross-talk between immature neuron-glia in neuroblastoma tumor cells upon induced differentiation.

Overall, our findings highlight how N- and I-type cells possess a similar differentiation potential, and represent an uncommitted stage between neuronal and glial fates, whereas S-type cells seem to be committed towards a glial lineage fate. These comparable characteristics display, however, a degree of variability within and amongst the N- and I-cell line subtypes suggesting a continuum process in the differentiation program reproduced by the cell lines.

## Conclusion

In summary, the characterization of each NB cell line subtype showed how all three cell lines represent an immature stage of the neural crest. However, both N- and I-type phenotypes are consistent with immature multilineage potential cells, since, upon induction, are able to progress towards neuronal as well as glial fates. By immunophenotype I-type cells did not show an intermediate pattern suggesting a distinct bipotential stage (neuronal and glial), although differential gene expression analysis revealed clear differences between the 3 subtypes. S-type cell lines, on the other hand, appear irreversibly committed towards glial lineage.

## Methods

### Tissue culture and cell lines

Phenotype characterization studies were performed on four N-type human neuroblastoma cell lines (LAN-1, LA1-55N, Be2-M17V and SH-SY5Y), four I-type cell lines (SK-N-Be2C, SK-N-JD, SK-N-LP and SK-N-ER) and three S-type (LA1-5S, SH-EP1 and SK-N-AS). All cell lines except SK-N-AS (Sigma, US), were kindly provided by Dr. B. Spengler, Fordham University, New York, NY; and Dr. NK. V. Cheung, Memorial Sloan-Kettering Cancer Center, New York, NY.

Cell lines were grown in RPMI high glucose media supplemented with 10% FBS, 2 mM L-glutamine and penicillin (100 U/ml) and streptomycin (100 μg/ml) (Reactiva, Spain) at 37°C in 5% CO_2 _atmosphere. Cell lines were never cultured more than 40 passages after thawing.

### Immunofluorescence

Cell lines were plated on round glass cover slips and incubated overnight at 37°C, then washed with PBS, fixed in 4% paraformaldehyde for 20 min, permeated with 0,1% Triton X-100 for 15 min, when required, and blocked for 30 min with 1% BSA. Primary antibodies, GD2 (BD, US), calcyclin (Sigma, US), NF68 (Zymed, US) Phox2b (Santa Cruz, US), GAP43 (Santa Cruz, US), ABCG2 (Alexis Biochemicals, Switzerland) and vimentin (Novocastra, UK) (Additional Table 1) were incubated for 1–3 h at room temperature or overnight (O/N) at 4°C and fluorochrome-linked secondary antibody was incubated for 45 min. Nuclear staining was obtained with 4'6-diamino-2-phenylindole (DAPI) 1:5000 in PBS (Sigma, US). Cells were mounted in Vectashield Mounting media (Vector Laboratories, US). Immunofluorescence was examined with a Leica epifluorescence DM5000B microscope (Leica Microsystems, US).

### Immunocytochemistry

Cell lines were cultured and fixed, permeated and blocked as explained above. Endogenous peroxidases were inhibited with H_2_O_2 _for 20 min. Primary antibodies S100 (Novocastra, UK), Sox10 (R&D systems, US) and c-kit (Zymed, US)(Additional file 1) were incubated for 15 min-O/N and chromogenically stained with Anti-mouse/rabbit Poly-HRP IHC detection kit (Chemicon, US).

### Differentiation assays

Differentiation experiments were performed as previously described [11]. Briefly, 5·10^5^–10^6 ^cells were harvested in T.75 culture flasks and 1 μM all-*trans*-retinoic acid (ATRA) (Sigma, US) or 10 μM 5-Bromo-2'-deoxyuridine (BrdU) (Sigma, US) were added to cell culture media to induce neuronal and glial differentiation, respectively. Treatment was maintained for 60 days by renewing media containing the drug three times *per *week. After 60 days, drug treatment was removed and cell cultures were maintained for 30 more days in media without drug. When treated with ATRA, cells were trypsinized at least once per week. For the two initial weeks, we also harvested different amounts of cells for immunofluorescence studies (2.5·10^5^–10^6 ^cells) and mRNA extraction (2.5·10^5^–3·10^6 ^cells). For the rest of time points we obtained the cells directly from the T.75 flasks.

Cell samples were harvested at days 1, 3, 5, 7, 15, 30, 60 and 30 days after treatment removal to analyze target markers. Control cell cultures, maintained without drug treatment, were harvested in parallel with drug-cultured cells. The experiments were performed in duplicate. See Additional file 2 for a flow-sheet diagram of differentiation experiments.

### Senescence analysis

Senescence of cells during differentiation experiments was analyzed by the senescence associated-β-galactosidase test kit (Sigma, US) based on Dimri protocol [42]. Briefly, cells were fixed in 2% glutaraldehyde/20% formaldehyde and then stained O/N with X-gal staining solution. Blue cells were considered positive. Non-treated cells cultured in parallel were used as control.

### Quantitative RT-PCR (qRT-PCR)

Quantification of transcript levels was performed for calcyclin, GD2 synthase, and SOX10. For GD2 synthase and calcyclin expression analyses all non-treated and differentiation induced cell lines LA1-55N, SK-N-ER and LA1-5S were used and, for SOX10 only the differentiation induced cell lines mentioned above were analyzed. RNA was isolated using Tri Reagent (Sigma, US), following manufacturers' protocols. cDNA was synthesized from 1 μg total RNA using random hexamers (Applied Biosystems, US) and M-MLV reverse transcriptase (Promega, US). q-PCR reactions (40 ng cDNA) and quantification, using the ΔΔC_T _relative quantification method, were performed on an ABI Prism 7000 Sequence Detection System using Assay-on-Demand Gene Expression products (Applied Biosystems, US). GD2 synthase primers and probes were described elsewhere [43]. Transcript levels were normalized to TATA box binding protein (TBP) expression values [44] and quantified relative to the cell line with the lowest amplification level or to each time course day non-treated control, in differentiation experiments. TBP coefficients of variation showed a low degree of variation amongst the cell lines and during the differentiation treatment (CV < 0.05). All experiments included no template controls and were performed in duplicate and repeated twice independently.

## Competing interests

The authors declare that they have no competing interests.

## Authors' contributions

SA, CL and JM are responsible for the initial conception and overall hypothesis of this study. SA, CL and JM are responsible for the design of this manuscript, including the original draft and subsequent revisions and design of this manuscript. CdT assisted with the initial concept and was involved with the draft and revisions of this manuscript; provided guidance for many of the experiments. SA and RP are responsible for the differentiation experiments design and immunocytochemical characterization of cell lines. SA and IG are responsible for the qPCR analyses, immunocytochemical characterization of differentiated cell lines. IG, ER and HB assisted with valuable technical assistance for experiments associated with this manuscript. All were also involved in the drafting and revisions for this manuscript. All authors read and approved the final manuscript.

## Supplementary Material

Additional file 1 **Flowsheet of differentiation induction protocol.** Click here for file

Additional file 2 **Primary antibodies used.** List of antibodies used for the immunostaining. Click here for file
