# Supplementary material for: Comprehensive characterization of neuroblastoma cell line subtypes reveals bilineage potential similar to neural crest stem cells
Source: BMC Dev Biol. 2009 Feb 12;9:12. doi: 10.1186/1471-213X-9-12 (PMC2647534; doi:10.1186/1471-213X-9-12)
Supplement: Additional file 1 — Flowsheet of differentiation induction protocol. [file 1471-213X-9-12-S1.ppt]

## Slide 1
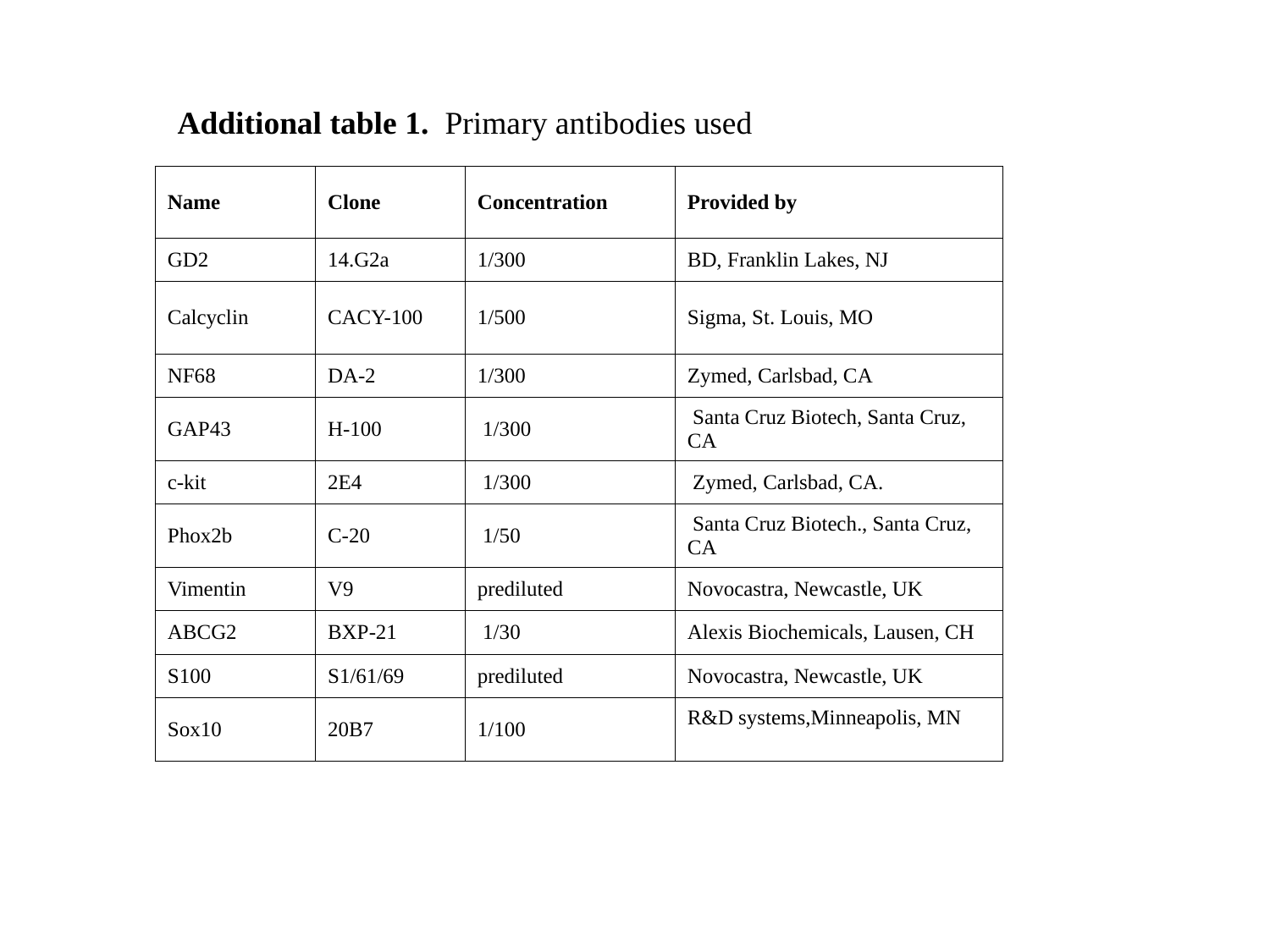

Additional table 1. Primary antibodies used
| Name | Clone | Concentration | Provided by |
| --- | --- | --- | --- |
| GD2 | 14.G2a | 1/300 | BD, Franklin Lakes, NJ |
| Calcyclin | CACY-100 | 1/500 | Sigma, St. Louis, MO |
| NF68 | DA-2 | 1/300 | Zymed, Carlsbad, CA |
| GAP43 | H-100 | 1/300 | Santa Cruz Biotech, Santa Cruz, CA |
| c-kit | 2E4 | 1/300 | Zymed, Carlsbad, CA. |
| Phox2b | C-20 | 1/50 | Santa Cruz Biotech., Santa Cruz, CA |
| Vimentin | V9 | prediluted | Novocastra, Newcastle, UK |
| ABCG2 | BXP-21 | 1/30 | Alexis Biochemicals, Lausen, CH |
| S100 | S1/61/69 | prediluted | Novocastra, Newcastle, UK |
| Sox10 | 20B7 | 1/100 | R&D systems,Minneapolis, MN |
