# Supplementary material for: Comprehensive characterization of neuroblastoma cell line subtypes reveals bilineage potential similar to neural crest stem cells
Source: BMC Dev Biol. 2009 Feb 12;9:12. doi: 10.1186/1471-213X-9-12 (PMC2647534; doi:10.1186/1471-213X-9-12)
Supplement: Additional file 2 — Primary antibodies used. List of antibodies used for the immunostaining. [file 1471-213X-9-12-S2.ppt]

## Slide 1
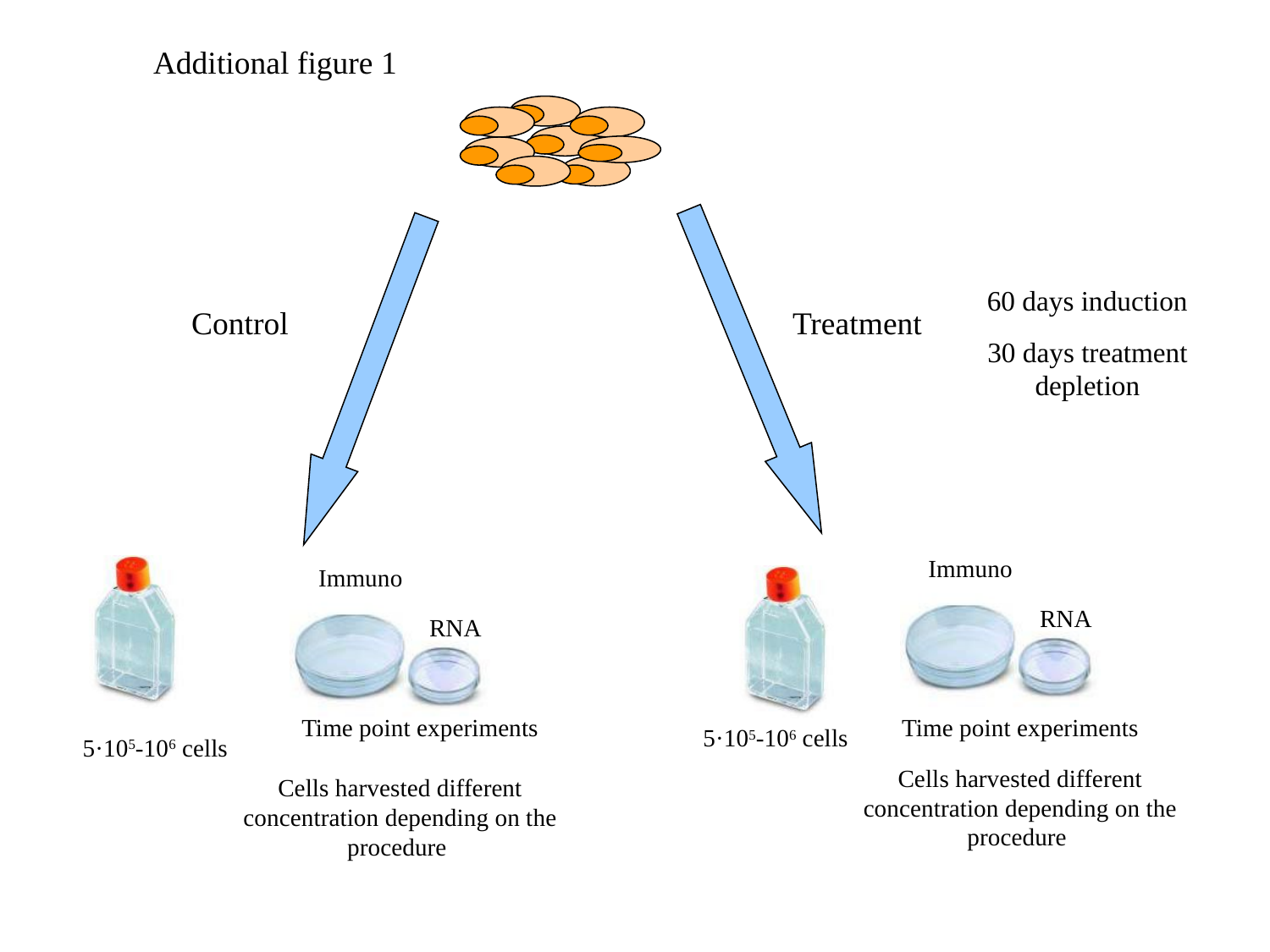

Additional figure 1
60 days induction
30 days treatment depletion
Control
Treatment
Immuno
Immuno
RNA
RNA
Time point experiments
Time point experiments
5·105-106 cells
5·105-106 cells
Cells harvested different concentration depending on the procedure
Cells harvested different concentration depending on the procedure
